# Application of Precision Technologies to Characterize Animal Behavior: A Review

**DOI:** 10.3390/ani14030416

**Published:** 2024-01-27

**Authors:** Abdellah Hlimi, Samira El Otmani, Fouad Elame, Mouad Chentouf, Rachid El Halimi, Youssef Chebli

**Affiliations:** 1 Regional Center of Agricultural Research of Tangier, National Institute of Agricultural Research, Avenue Ennasr, BP 415 Rabat Principale, Rabat 10090, Morocco; 2 Laboratory of Mathematics and Applications, Faculty of Science and Technology, Abdelmalek Essaâdi University, Tangier 90000, Morocco; 3 Regional Center of Agricultural Research of Agadir, National Institute of Agricultural Research, Avenue Ennasr, BP 415 Rabat Principale, Rabat 10090, Morocco

**Keywords:** behavior, management, precision livestock farming, ruminant, tracking

## Abstract

**Simple Summary:**

The information that can be deduced from animal behaviors is diverse. Unlike in the past, these behaviors can now be monitored for extended periods of time, thanks to the many advanced tools and sensors. The changes in behavioral patterns can provide many indications and clues about various aspects of the animals’ needs and status. In this review, we evaluate three types of technology used to identify the behaviors of three types of ruminants. These tools have the potential to significantly assist farmers in the continuous development of their practices.

**Abstract:**

This study aims to evaluate the state of precision livestock farming (PLF)’s spread, utilization, effectiveness, and evolution over the years. PLF includes a plethora of tools, which can aid in a number of laborious and complex tasks. These tools are often used in the monitoring of different animals, with the objective to increase production and improve animal welfare. The most frequently monitored attributes tend to be behavior, welfare, and social interaction. This study focused on the application of three types of technology: wearable sensors, video observation, and smartphones. For the wearable devices, the focus was on accelerometers and global positioning systems. For the video observation, the study addressed drones and cameras. The animals monitored by these tools were the most common ruminants, which are cattle, sheep, and goats. This review involved 108 articles that were believed to be pertinent. Most of the studied papers were very accurate, for most tools, when utilized appropriate; some showed great benefits and potential.

## 1. Introduction

The use of grasslands and forest pastures is an ancestral practice. They are mainly utilized for grazing by ruminants. In fact, this is a consistent aspect of different production systems, especially when there are sufficient pastoral resources. This will ensure the fulfillment of most of their daily needs [1], either in the pastures or on the farm. Animals need feeding until fulfilled, but how do we understand and address their hunger, fatigue, thirst, and sickness?

Animals are complex creatures; they cannot convey in words their needs; nevertheless, different studies have shown that they have patterns that they follow, and variations in these patterns have meaning [2,3,4], which could give insight into their needs, preferences, and other physical and biological states [5]. This highlights the importance and the merits of studying their behaviors.

Research on behavior and activity budgets in different livestock systems has been carried out for a long time. More than forty years ago, Anderson and Kothmann analyzed cattle travel and the factors that could affect it using pedometers [6]. Since then, many studies have been carried out on diverse behaviors, such as basic daily activities (walking, resting, lying, etc.) [7,8,9]; feeding activities [10,11]; welfare [12]; lameness [13]; estrus and parturition detection [14,15]; livestock distribution [16]; sexual actions [17]; and nursing [18]. These studies and others have elaborated upon different types of monitoring devices, because there is a limit to what the herder can detect and monitor regarding animals’ behaviors. As is known, the visual approach is extremely subjective, and it is a very limited and time-consuming method [19]. It is also worth noting that visual observation is the oldest approach to monitoring animals’ behaviors. To this day, it is still used, but mainly in the validation of data recorded by other precision and smart tools [20].

Precision livestock farming (PLF) refers to the use of several technologies to manage and optimize various aspects of livestock farming [21]. PLF involves applying process engineering principles and techniques to livestock farming, enabling the automated monitoring, modeling, and management of animal production [22]. A large set of sensors are used in a separate or combined way [23]—for example, accelerometers [24], global positioning systems (GPS) [25], unmanned aerial vehicles (UAVs) [26], cameras [27], smartphones [28], microphones [29], tags [30], and ruminal boluses [31]. These tools have distinct operating instructions; they could be wearable on different parts of the body, static or movable, or inserted into the animal itself.

This review attempts to describe the development and application of the most commonly used technologies and their potential: wearable sensors—more precisely, GPS and accelerometer-based sensors—video observation tools such as drones and cameras, and smartphones. These technologies offer the most benefits because they can monitor the majority of animal behaviors. This study focuses on three types of ruminants, cattle, sheep, and goats, as they are the most common in livestock production systems around the world.

## 2. Methods

The methodology of the research in this systematic literature review was based on the Preferred Reporting Items for Systematic Reviews and Meta-Analyses (PRISMA) guidelines [32]. The bibliographic research was conducted in the month of July 2023 using different combinations of the following keywords: livestock, goat, cattle, sheep, precision farming, precision livestock farming, sensor, GPS, accelerometer, pedometer, icetag, eartag, smartphone, drones, video observation, video monitoring, PLF, UAV. The databases used in the search were mainly Google Scholar, Science Direct, PubMed, and Scopus. Mendeley was also used in managing the references and organizing our research.

In this study, the selection criteria were as follows:

▪ Only peer-reviewed articles and conference papers were selected;
▪ Studies had to be within the research objective;
▪ The literature had to be in English;
▪ The full text study had to be accessible;
▪ Cattle, sheep, and goats were taken into consideration;
▪ The technologies of sensors, video observation, and smartphones were considered.

**Figure 1:**
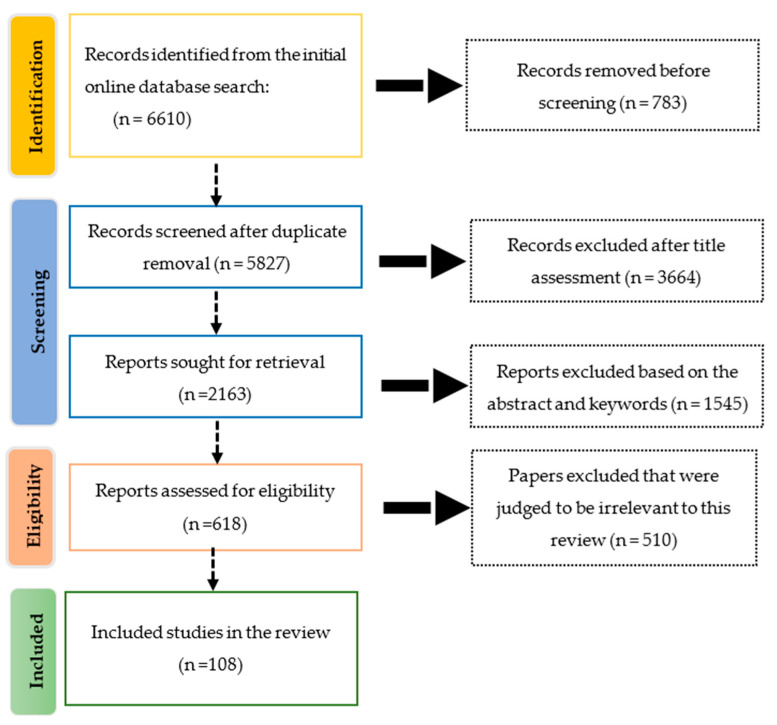
Flowchart of the systematic review process.

After the initial online database search, 6610 studies were obtained, which, after duplicate removal, were reduced to 5827. In the screening phase, based on titles, 3664 documents were excluded because they were about other animals or other technologies beyond the scope of this study. Abstracts and keywords were then examined, leaving 618 articles for further analyses, where the complete texts were checked to determine whether they satisfied the objective criteria. Ultimately, 108 articles were judged to be relevant to this review. The procedure of the filtration of the papers is summarized in Figure 1, based on the PRISMA guidelines.

## 3. Wearable Devices

In this section, two common tools were discussed: accelerometers and GPS-based sensors. The accelerometer monitors one of the most important aspects of the animals’ behavior, namely their acceleration, while GPS is capable of tracking their continued movements and locations, even when they are out of sight. This enables managers or farmers to know their exact position at any given time. There are many precision tools, like accelerometers, pedometers, GPS, and other motion sensors, which are used to monitor animals’ movements [33]. Recently, several devices equipped with accelerometers have been developed, such as SenseHub. The SenseHub monitoring technology is a complex yet user-friendly animal monitoring solution. It includes sensors to monitor vital signs or detect signs of illness. It allows for the unique identification of each tagged animal. This helps farmers to detect several parameters related to animal welfare, reproductive performance, health, and the nutritional status of individual animals and groups. The prospect of combining the accelerometer and GPS in studying animals’ behaviors will be discussed.

Sensors in general need to be attached correctly with the appropriate positioning, aiming to optimize the results by taking into account the animal’s well-being [34]. They are attached to some part of the body while aligned with its axes. This direct attachment prevents the sensor from moving independently of the animal, which may improve the recorded results [35].

### 3.1. Animal Activity and Behavior Using Accelerometers

As the name implies, the accelerometer is a device that measures acceleration, which is, by definition, the change in velocity over time [36]. Their utilization in monitoring animal behaviors started in the previous decade [36], and many studies have used this device to evaluate the physical activities of different species. The accelerometer can be distinguished based on the number of used axes, such as a 1-axis accelerometer that measures acceleration in a single direction, i.e., up or down; a 2-axis accelerometer that measures acceleration in two perpendicular directions, typically up/down and left/right; and a 3-axis accelerometer that assesses acceleration in all three dimensions, providing a more comprehensive view of the movement in 3-dimensional space [37]. This can help to sense orientation, coordinate acceleration, vibration, shock, and falling in a resisting medium [37]. In its application in monitoring animals, this tool could be attached to different parts of the body [38], and it can detect distinct changes and variations in the animal’s patterns and status [39]. The studies discussed in this review using accelerometers were mostly performed in the United States and Europe (Table 1, Table 2 and Table 3). Other countries have shown interest in this field of research, such as Australia, New Zealand, China, and Brazil.

#### 3.1.1. Cattle Behavior

Accelerometers are mostly used in recording animals’ daily activities, such as walking, ruminating, and lying, at different times of the day, seasons, and conditions for different species, with each study focusing on a set of behaviors, aiming to be as precise as possible. Studies tend to validate their results using visual observation by experts. Results usually vary from moderately to highly accurate. Cattle and the changes in their behavior have been the topic of several studies. Riaboff et al. [40] were successful in predicting cattle behaviors (grazing, walking, ruminating lying, ruminating while standing, resting while lying, and resting while standing) using a neck-collar accelerometer in a pasture-based system, with accuracy of 98%. Rumination behavior was monitored with an ear-tag accelerometer in a semi-enclosed barn. This behavior was detected with 98.4% accuracy [41] (Table 1a). Another type of behavior was monitored with a neck-collar and ear-tag accelerometer in an intensive system, which considered licking, where the overall performance of both types was acceptable (88 and 98% in accuracy) but with a small advantage in favor of the neck collar [42]. Calves’ behaviors were also monitored, such as suckling behaviors. Kour et al. [18] successfully identified and estimated more than 95% of suckling bouts and durations in a pasture-based system.

#### 3.1.2. Cattle Health and Welfare

Another important matter is cattle health and welfare. Jaeger et al. [43] aimed at assessing cattle’s welfare under a normal production system (rotational grazing scheme) with an ear-tag accelerometer, which was found to be impacted by many factors, such as hygiene, aggressiveness, basic behaviors, and intra-herd rank. Lameness can affect cattle behaviors according to Thorup et al. [19], who used a leg-mounted accelerometer to prove that lame cows in intensive systems tend to spend more time lying down and less time walking. In a rotational grazing system, Tobin et al. [12] aimed at detecting illness before symptoms appeared, which was successful as they noticed a movement decrease in ill heifers 24 h previously. On the other hand, Sutherland et al. [44] considered diarrhea prediction with neonatal calves, noticing changes in behavior 4 days before the diagnosis.

#### 3.1.3. Cattle Reproduction: Estrus and Calving

The accurate detection of estrus and calving is very important for farmers; many researchers have attempted to predict them and observe changes in these periods. Benaissa et al. [45] used several combinations of accelerometers in a free-stall barn environment to detect estrus. This method was successful but was more accurate in the case of using one sensor on the same animal. A sudden behavior change could also indicate the time of calving. Borchers et al. [46] monitored cattle behaviors in a pasture-based environment and precisely detected these changes with sensitivity and specificity of more than 80%.

#### 3.1.4. Accelerometer Accuracy

Comparing accelerometers’ accuracy, or even the validation of new ones by tested devices, has been the subject of numerous studies. In an intensive system, Borchers et al. [47] compared six commercially available accelerometers for different behaviors (lying, feeding, and rumination). Each sensor type was better adapted to some studied behaviors than others (Table 1a). An ear-tag accelerometer for the monitoring of calves’ drinking behavior was evaluated under an intensive system with accuracy of 96.2%. The early detection of changes in this behavior would prevent complications [48] (Table 1a). A noninvasive accelerometer for the monitoring of cattle sleep, attached to the harness, was evaluated, which was very accurate (92.2 ± 0.8%) [49]. The sensor’s position is also a variable that may affect its performance. Aloo et al. [50] studied this factor by placing the device between the dewlap, leg, and harness. They found that the latter was the most adequate. Van Erp-Van Der Kooij et al. [51] compared leg and neck placement and found a good correlation for both sensors for the studied behaviors (with a correlation coefficient of >0.85) except walking.

#### 3.1.5. Sheep Activity and Behavior

Sheep have been the subject of several extensive studies of behavior (Table 1b). Ikurior et al. [52] monitored sheep’s common behaviors using different accelerometer placements in an extensive system, and the overall accuracy was 89.6% for grazing, walking, and resting. Some specific behaviors may also be of interest, such as lying behaviors, which were successfully monitored by a leg-mounted accelerometer in an extensive system, and it has been concluded that many factors can affect sleep, namely sex, age, weight, and pregnancy [9]. Lamb suckling behaviors were investigated by Kuźnicka and Gburzyński [53] using a neck-mounted accelerometer; the detection rate was 95%. The monitoring of behaviors in relation to the diet, like chewing and biting activities, was achieved in a pasture-based environment, with sensitivity for biting and chewing activity (95.5% and 93%, respectively) improving as the time interval increased [54]. Monitoring the herd as a whole, by monitoring some animals and then using the data to predict or deduce the others’ behaviors and classify their behaviors with a neck-mounted accelerometer in a rectangular field, had a success rate of 74.8% [55], which is reasonable given that it is not possible or convenient to study every animal in the herd.

An important task is to evaluate the effectiveness of the sensor on sheep. A comparison between three types of accelerometers with variable configurations was conducted in a pasture-based environment. It was found that behaviors were successfully identified, with the best performance for the ear-mounted device (86% to 95% accuracy) [56]. Another sensor was evaluated to determine which behaviors could be detected easily. In a semi-improved pasture, the authors placed the sensor under the jaw and found that the detection of grazing behavior was the easiest. Another factor that alters the results is the placement of the sensor. Decandia et al. [57] considered three different placements (mouth, nape, and neck) under an extensive system. The neck-mounted device had the best results (90% of accuracy). Various sensors were also evaluated in recording some specific behaviors—for instance, urination events—with an accurate estimation rate of 100% [58], or lameness with three different placements (leg, ear, and neck), with the best results (87% of accuracy) for the leg deployment [13].

In the livestock industry, most research seems to be focused on reproduction and, therefore, parturition and lambing. Gurule et al. [59] studied the variations in ewes’ behaviors around parturition, in an intensive system, and achieved the monitoring of activity with 87.2% accuracy; the system was very helpful in predicting the approach of lambing. A similar study, but in an extensive system, showed that, around lambing time, grazing decreased in favor of other behaviors, such as lying and being active [60]. Concerning the sexual activities of rams, in a pasture-based environment, mounting and service detection were monitored successfully, with overall sensitivity of 77.9% [17].

Accelerometers, as sensors, offer valuable insights into various aspects of ruminant behavior, health, and management; nevertheless, it is important to mention certain challenges associated with accelerometer use, such as the data processing complexity, device attachment considerations, and battery life (Table 4). Farmers, as end users, need to approve and accept the technology, which, in general, can be complex as they tend to prefer the visual approach [61]. According to a study by Van De Gucht et al. [61], farmers were reluctant to use an automatic lameness detection system, but this changed when they were informed about the serious consequences of lameness. Farmers’ interpretation and evaluation of data is a strategic procedure that aims to lead to informed decisions and improve overall livestock management. Key steps include ensuring the accuracy and quality of various sensor data, understanding the key capabilities of PLF technology, and setting benchmarks and goals for performance improvement. Farmers analyze patterns and trends over time, identify correlations between variables, and integrate PLF data into their farm management systems. Using PLF data to support decision making, farmers can adapt their practices, such as reproduction and welfare practices, while continuous training ensures effectiveness. A commitment to constant improvement with regular evaluation is necessary to be able to adapt to future demands.

### 3.2. Animal Tracking Using GPS

A GPS sensor is a device that can be placed on different parts of the body to track and record animals’ real-time locations, and thus their movement, especially in large pastures [38]. This information on the animal’s position could provide details about the topography, vegetation type, water source locations, grazing locations, calving sites, and temperature [1,62]. GPS is especially useful in large pastures. The position and other data are communicated to a user’s server via global satellites. Therefore, one of its main uses is in monitoring livestock behaviors under pasture conditions. Several authors [63,64,65] have studied different aspects of cattle behavior. Castillo-Garcia et al. [25] evaluated the sheep’s grazing effects on vegetation to determine whether they were beneficial or not for pastures. 

The cattle diet was monitored by Orr et al. [66], by tracking their preferred paths. They concluded that cattle favored shorter, easy-to-digest material during the day, while they selected material with higher crude fiber in the evening.

Social interaction with other species was examined by Brown et al. [67]. They studied the influence of the presence of cattle on the behavior of bighorn sheep. The latter stayed vigilant in the presence of cattle, with a noticeable decrease in foraging bite rates.

Ganskopp [3] manipulated the water and salt distribution, in a very large pasture, to determine which was more important to cattle. He found that water was more important, as they shifted towards it whenever they moved, which can be a very effective way to alter the cattle distribution with minimal interference.

There are also studies interested in developing GPS-based systems, such as Halasz et al. [68], who provided near-real-time monitoring with a constructed GPS tracking collar. 

These studies and others clearly show the limited potential of GPS. Position data are indeed very helpful in many aspects but combining them with other sensors will allow their full potential to realized. This may include accelerometers, which will be the subject of the next section.

### 3.3. Accelerometer and GPS Sensor Combination

The combination of accelerometers and GPS increases the accuracy and sensitivity of detailed animal behavior detection [62]. Combining positioning data with other sensors, in general, would provide higher prediction accuracy as different behaviors emerge in different locations. Even with the same movement patterns, the animal’s real behavior could be deduced [69]. The purpose of the combination of different sensors is to obtain higher accuracy in detecting behaviors. For instance, Cabezas et al. [70] successfully classified cattle behaviors with a GPS and accelerometer integrated sensor attached to the neck in a pasture-based environment. The results were quite accurate, with grazing having the highest accuracy (93%) and ruminating (88.1%) having the lowest.

Goat behavioral classification, in an extensive system, was performed during different seasons with this combination, which confirmed that this ruminant tends to spend more time grazing during the spring but travels greater distances during the summer and autumn [23] (Table 1c). The set of data collected from two types of sensors may lead researchers to unexpected results, like Tobin et al. [71], who concluded, while surveying water malfunctioning based on cow behaviors, that the ones that experienced water shortages due to failure tended to stay closer to the water source.

This type of association between sensors is not fully understood. Researchers are still evaluating and testing their optimal use. Sprinkle et al. [72] tested this combination in a pasture-based environment, and patterns of grazing behaviors were accurately identified. In another study, the authors constructed a GPS collar combined with a three-axis accelerometer, and they tested this tool on steers. They had accurate results concerning grazing locations and timings, which tended to be during the morning and evening for 8.67 to 10.49 h per day [73].

There are also some studies of specific behaviors, such as Barker et al. [74], who developed a position- and activity-based system that detected lame cows successfully (accuracy ranged from 80.8% to 94.2%) based on changes in normal behavior. Another application was developed to detect sheep’s parturition. Fogarty et al. [15] used a global navigation satellite system (GNSS) tracking collar and an accelerometer ear-tag in their study. They had moderate accuracy at first, but it could be increased to 91% if an earlier false alert was permissible.

The combination of accelerometers and GPS results in a synergistic relationship that exploits the strengths of both sensors to provide a good understanding of ruminants.

**Table 1:** (**a**) Studies on cattle behavior and activity budget in different livestock systems. (**b**) Studies on sheep behavior and activity budget in different livestock systems. (**c**) Studies on goat behavior and activity budget in different livestock systems.

(**a**)					
**Aim**	**Technology**	**Livestock System**	**Country**	**Main Result**	**Reference**
Behavior	Accelerometer	Intensive	United Kingdom	Accuracy of 83% in classifying behavior	[24]
			Australia	Accuracy of 88% to 98% in monitoring licking behavior	[42]
			Australia	4-month-old calves suckled fewer times, but for longer	[73]
			United Kingdom	Classification of rumination, eating, and other behaviors with precision of 0.83	[74]
		Pasture-based	France	The accuracy of prediction of the main behaviors was 98%	[40]
		Semi-enclosed barn	United States	Accuracy of rumination detection was 86.2%	[41]
		Three dairy farms	Italy	Accuracy of behavior detection was 85.12%	[75]
		Dairy farm	Italy	Accuracy of classifying behavior was 96%	[76]
	GPS	Extensive	United States	Cattle followed water more than salt	[3]
			Hungary	Weather fronts affected the herd’s route	[64]
		Pasture-based	Malaysia	Observation of the grazing patterns was accurate	[63]
			England	Cattle tended to favor shorter material during the day and material of higher crude fiber in the evening	[66]
		Commercial farm	Spain	Sensor was able to detect hotspots of dung deposition	[77]
	GPS-GPRS	Extensive	Spain	Distance traveled daily was 3147 m	[65]
	Accelerometer, GPS	Pasture-based	Australia	Description of the animals’ movement and some behaviors was successful	[78]
			Spain	Accuracy of classification of behavior was 93%	[70]
	Accelerometer, RFID	Pasture-based	Australia	Accelerometer correlated highly with the observed duration of drinking events	[79]
	Accelerometer, magnetometer	Intensive	Tasmania	Grazing, ruminating, and resting were identified accurately	[80]
	Accelerometer, cameras	Intensive	China	Accuracy of 94.9% in recognizing behavior	[81]
Sensor evaluation	Accelerometer	Intensive	United States	The correlation was high between results of the sensor and visual observations in monitoring behavior	[7]
			Australia	Heavy breathing detected by the sensor correlated well with visual observations	[82]
			Japan	Precision of classifying behavior was 99.2%	[83]
			Germany	Accuracy was 70.8% in monitoring selected behaviors	[84]
			Germany	Accuracy was 96.2% in monitoring drinking behavior	[44]
			United States	Each sensor had high correlation with visual observations for a specific behavior	[43]
			United States	Accuracy was over 92.2% in monitoring sleep	[45]
			Netherlands	The sensor had a correlation of over 0.85 with the visual observation in monitoring behaviors	[47]
			Netherlands	Sensor’s results and visual observations correlated well for monitoring of behavior	[85]
			Netherlands	Sensitivity was over 96.1% for monitoring of behavior	[86]
		Extensive	Brazil	Over-sampling increased accuracy in prediction of grazing behavior	[87]
			Kenya	The harness was more accurate	[46]
		Pasture-based	United States	RumiWatch had accurate results for the studied behaviors	[20]
			Ireland	MooMonitor+, RumiWatch, and visual observation had high correlation for measurement of grazing behavior	[88]
			Australia	Accuracy was 95% to 98.8% in measuring suckling behavior	[18]
			Germany	Rumination and eating behavior were monitored accurately	[89]
			Australia	Grazing, resting, and ruminating were accurately detected	[90]
		Loose-house system	Denmark	The AfiTagII correlated very highly with direct observations and IceQube recordings in monitoring lying behavior	[91]
		Housed in an outdoor dirt floor pen	Canada	Sensitivity and specificity were 95% and 76% for feeding and 49% and 96% for rumination	[92]
	GPS	Pasture-based	United States	The Clark ATS provided real-time tracking	[68]
	Pedometer	A 0.2-ha sown pasture	Japan	Correlation coefficients between the pedometer values and the number of bites were all over 0.9	[8]
		Pasture-based	United States	Distance traveled increased with larger pasture	[6]
	Accelerometer, GPS	Intensive	United Kingdom	Accuracy was 80.8% to 94.2% in detecting variations in feeding behavior	[93]
		Pasture-based	United States	Patterns of behavior were accurately identified	[72]
			United States	Time spent grazing from 8.67 to 10.49 h daily	[94]
	Accelerometer, pedometer	Extensive	Italy	Accelerometer and direct observations for ruminating, feeding, standing, and lying correlated well	[95]
Health and welfare	Accelerometer	Intensive	New Zealand	Change in behaviors began 4 days before the diagnosis	[49]
			Denmark	Lying duration increased by 40 min but walking decreased for lame cows	[19]
		Intensive system with constant access to pasture	United States	The diseases had negative effects on ruminating and walking	[96]
		Rotational grazing system	Australia	24 h before the symptoms, heifers moved less	[12]
		Pasture-based	Germany	Associations found between sensor behavior traits and monitored cow behavior	[48]
	Pedometer	Individual pens (3 m^2^) in a calf barn	United States	Activity drop before the diagnosis	[2]
Estrus and calving	Accelerometer	Pasture-based	United States	100% sensitivity, 86.8% specificity in detecting changes in behavior	[51]
			New Zealand	Monitoring of behavior was successful	[97]
		Free-stall barn environment	Belgium	Performance increase with more sensors	[50]
		Lactating cows were housed in 2 free-stall pens	United States	Sensors were at least as successful as visual observation in detecting estrus	[98]
	Pedometer, accelerometer	Dairy cattle farms	Germany and Italy	Estrus detection was accurate	[99]
	GNSS	Commercial farms	Spain	Sensor provided indicators on the occurrence of calving	[100]
	Accelerometer, GNSS	32 ha paddock	Australia	Accuracy of 98.6% in calving detection	[101]
Bite rate	Accelerometer	Intensive	Australia	Semi-supervised linear regression model predicted bite rate well	[102]
(**b**)					
**Aim**	**Technology**	**Livestock System**	**Country**	**Main Result**	**Reference**
Behavior	Accelerometer	Extensive	New Zealand	Accuracy of 89.6% for grazing, walking, and resting	[52]
			Wales	Accelerometers correlated perfectly with video observations for lying behavior	[9]
			Poland	Suckling episode detection rate of 95%	[53]
		Pasture-based	Australia	5 s time interval was best in identifying biting and chewing	[54]
		A rectangular field of 110 × 80 m	Denmark	Classification of behavior success was 74.8% for the entire herd	[55]
		Sheep alternating between intensive and extensive system	Italy	Accuracy of 93% in prediction of bite rate	[103]
	GPS	Extensive	Canada	Livestock’s presence had an effect on bighorn sheep’s behavior	[67]
	Accelerometer, gyroscope	Three pasture paddocks of 72 m^2^	Australia	Behavior classification had accuracy of 87.8%	[104]
Sensor evaluation	Accelerometer	Extensive	Italy	Collar attached was the best with accuracy of 90%	[57]
			Wales	100% accuracy for urination events	[58]
			Australia	Accuracy was best (87%) for the leg deployment	[13]
		Pasture-based	Australia	Ear-mounted sensor was the most accurate with 86% to 95%	[56]
		Semi-improved pasture for the 1st study and a small pen in the 2nd study	Australia	Grazing behavior was the easiest to detect	[105]
		Pasture-based but they were gradually removed from pasture	Italy	The device performed well and the number of bites was accurate	[106]
Parturition and sexual activity	Accelerometer	Intensive	United States	Accuracy of behaviors was 66.7%, and that for activity was 87.2%	[59]
		Extensive	New Zealand	Ewes were more restless around parturition	[60]
		Pasture-based	Spain	Sensitivity for mounting detection was 77.9% and for service detection was 94%	[17]
	GNSS logger, accelerometer	Extensive	New Zealand	Detection of parturition events and lambing activity was accurate	[15]
Effects of grazing on vegetation	GPS	Extensive	Spain	Grazing, depending on its intensity, may benefit or not the pastures	[25]
Health and welfare	Accelerometer	5.5 ha paddock	Australia	Accelerometer-based sensor can identify changes in sheep activity associated with *H. contortus* infections	[107]
(**c**)					
**Aim**	**Technology**	**Livestock System**	**Country**	**Main Result**	**Reference**
Behavior and activity	GPS, accelerometer	Extensive	Morocco	Sensors monitored accurately the grazing activities of dairy goats	[1,108]
			Morocco	Sensors monitored accurately the grazing activities of meat goats	[23]
		Pasture-based	Germany and Oman	Recognition of eating 87% to 93%, 68% to 90% for resting, and 20% to 92% for walking	[109]
	Accelerometer, gyroscope	Extensive	Argentina	Prediction of behaviors had precision of 85% and recall rate of 84%	[110]

GPS, global positioning system; GPRS, general packet radio service; RFID, radio frequency identification; GNSS, global navigation satellite system; the number of animals (cattle) in the research varied between 3 and 348; the number of animals (sheep) in the research varied between 1 and 96; the number of animals (goats) in the research varied between 1 and 8; Intensive system, continuous supplementation of animals by cereal-based feed or industrial supplements is the standard; extensive system, mainly involves small ruminants and resource-constrained breeders, depending on rangeland; pasture-based, a system that relies significantly on pastures, which include grasses, legumes, and herbs [111].

## 4. Video Observation

The devices used in video observation are usually stationary sensors [38]. A camera can be placed in a location where it can record important behaviors. However, it would only capture a small portion of the animal’s daily activities and disregard the rest [62]. Furthermore, it is not applicable in large pastures and extensive systems, which makes UAVs more useful [62]. They can follow a herd with an attached camera and record most of their behaviors in pastures. Data collected from cameras, either stationary or mobile, are useful in different aspects, such as examining the behavior and position or for the counting of animals (especially large herds over vast pastures) [62]. Each type of video observation will be discussed further (Table 2).

### 4.1. Stationary Camera

The invention of photography or cameras dates back to the 1800s. Animal enthusiasts began to employ this device to observe wildlife at the end of 19th century, as it evolved into a much smaller, portable, and easier-to-handle tool [112]. This tool has experienced rapid development up to the present, where it is frequently used in livestock monitoring in farm environments [113] or pastures [27]. This is a type of sensor that is considered non-invasive, and it can monitor animals’ feed and water intake and social interaction and provide numerous other valuable types of data in real time [114]. Cameras are deployed around animals’ most frequented locations, especially in barns, to capture different views. From a financial point of view, the use of a camera is very cheap, as one camera can observe multiple animals, if not the entire herd. In addition, there is no deployment or recovery of sensors from the animals [115].

Cattle behaviors (standing, lying, feeding, drinking, and walking) were observed by Mitlöhner et al. [116] in an intensive system, using different intervals for scan sampling (Table 2). The shorter ones were determined to be precise for all the behaviors except drinking and walking. Time sampling was not precise, and focal animal sampling was accurate for most behaviors. Another study [113] monitored cattle in an intensive system and successfully recognized as many as 15 types of behavior (walking, standing, resting, eating, sleeping, standing up, lying down, self-grooming, fighting, feeding, social licking, mounting, ruminating, moving head, and moving tail).

Cattle health was studied by Kang et al. [4]. They used a camera fixed on a tripod 6 m from the side of a passing alley to monitor lameness by detecting when an animal’s weight was not supported equally by the hooves, and the accuracy for lameness was 96%.

A bird’s eye video camera was used to study cattle’s breeding conditions in the pasture while monitoring their interactions. This method showed great prospects, and small adjustments could improve it even more [27]. Lastly, the interaction between cattle and elk was observed [117]. The latter avoided contact with cattle while staying close to the water stream and when cattle spread wider [117].

The utilization of stationary cameras in monitoring livestock stands as a simple and easy-to-implement technology that offers a plethora of advantages—in particular, providing a stable and continuous method of animal surveillance.

### 4.2. Unmanned Aerial Vehicles

UAVs, commonly known as drones, are, as the name suggests, flying vehicles without a pilot that are controlled remotely and have a diverse set of applications (civilian, military, agricultural, etc.). In the livestock domain, with an attached camera, drones are automatic and programmed tools used to survey the flock [118], especially animals in distant pastures [119]. They can achieve continuous monitoring by tracking and photographing the animal’s behaviors [120], and their results are considered more reliable than those of other sensors. They can track animals on vast farms or in pastures, inspect feed and water availability, count animals, and even analyze their current health status if equipped with advanced tools such as thermal sensors [121]. However, if drones are handled by an untrained user, they may harm animals by causing stress (for example, by flying drones close to the herd) [62].

The monitoring of animals using UAVs was studied by Vayssade et al. [122] within goats. Animals were identified with sensitivity of 74%, while activity detection reached 78.3%. The spatial distribution of cattle and yak was observed by Mufford et al. and Sun et al. [16,123], respectively. Related cattle tended to stay close to each other, while the yak’s spatial distribution varied depending on numerous factors like the season, time of day, and location.

Another application is the counting of animals. Wild animals and livestock grazing on the Tibetan Plateau were monitored, resulting in a complete census. The ratio of large herbivores to livestock was 1:4.5 in sheep units [124]. Cattle can be monitored under harsh conditions [125], under different production systems [126], and even by evaluating new methods of counting and detection [127]. Many other studies have aimed at improving the drone’s performance through different approaches, including attempting a method for sheep detection from different altitudes [26], increasing the drone’s covered area by using a tilted angle [128], and assessing their applicability in an enclosed environment [129] (Table 2).

It is clear that drones have great potential, with their versatile roles in surveillance, data collection, and management, but there is room for improvement to address the current limitations, namely thick vegetation and night restrictions [62].

**Table 2:** Studies on behavior and activity detection in animals using video monitoring.

Species	Technology	Aim	Livestock System	Country	Main Result	Reference
Cattle	Camera	Behavior recognition	Intensive	South Korea	15 different types of activity were accurately recognized	[113]
		Behavior in feedlots		United States	Scan sampling with short intervals correlated highly with continuous observation; time sampling was not accurate; and focal animal sampling was accurate for most behaviors	[116]
		Lameness detection		China	Correlation between lameness and the supporting phase was 0.864	[4]
		Change in behaviors around calving		Italy	The frequency of lying, tail raising, and walking increased during the pre-calving period	[130]
		Tracking under farm conditions		South Korea	Accuracy of 84.49% in tracking cattle	[131]
		Indentifying and recognizing activities		Italy	Detecting and recognizing cattle was effective, with mean average precision of 89%	[132]
		Temporal and spatial use of riparian pasture	Semi-extensive	United States	Elk traveled within the stream channel while grazing. Cattle drank from the stream but did not enter it and tended to lie away from the channel	[117]
	Bird’s eye camera	Breeding conditions	Semi-extensive	Japan	Cattle’s detection accuracy was improved by the proposed method	[27]
	UAV	Drones’ usage in intensive systems	Intensive	Netherlands	Usage of drones for indoor livestock management was successful	[129]
		Counting and detection	Extensive	Brazil	Cattle counting was a success, especially with reduction in duplicate counting	[127]
		Counting		Brazil	Accuracy exceeded 90% in counting cattle	[125]
		Increase the covered area by the UAV		Brazil	Oblique images were successful under some conditions	[128]
		Monitoring yak’s spatial distribution		China	This method of monitoring the yak’s herd was successful	[16]
		Monitoring animal distribution	Semi-extensive	Canada	Related pairs were closer than non-related ones	[123]
		Counting under different production systems	Extensive and intensive	Australia	The proposed system accurately classified cattle with accuracy of 96%	[133]
Sheep		Detect livestock from images	Semi-extensive	New Zealand	Sheep detection at 80 m was better than at 120 m	[26]
Goat		Animal monitoring		France	Animal detection had sensitivity of 74% and activity detection had 78.3%	[122]
Wild animals and livestock		Estimation of feed quantities of animals	Extensive	China	The population census was successful, with a large wild herbivore to livestock ratio of 1:4.5 in sheep units	[124]

UAV, unmanned aerial vehicle.

## 5. Smartphones

Over the last decade, there has been a rapid increase in smartphone ownership. Access to the internet and the use of smartphone applications around the world, in both urban and rural regions [134], has led to a rise in interest in developing smartphone applications to support farmers in herd management [135]. Researchers have investigated the feasibility of monitoring animals hourly based on smartphone data [114], as well as their compatibility with other sensors to allow the rapid intervention of farmers when needed [136].

Using old smartphones as sensors to monitor livestock behavior is an interesting strategy that can represent a feasible and cost-effective solution for the observation of some behaviors or for data collection. There are various ways that they can be used for PLF, such as GPS functionality (they can be used to track the locations of livestock or mark specific points on the farm); to record data manually, such as feeding schedules; to communicate among farm members to share updates and coordinate tasks; to be informed about current weather conditions; and finally to set reminders on the phone for important tasks, feeding times, or health check-ups for the livestock.

Nowadays, smartphones tend to be equipped with many sensors, such as accelerometers, gyroscopes, GPS, and magnetometers [137]. The functions that these sensors provide can be applied to record an animal’s location, behavior (rumination, eating, etc.), or any other needed data. It can also allow easy access to advice about herding practices, for example, or any other related matter [134]. Farmers can keep track of animals’ data by recording them on their phones. This practice, if done collectively, can be used to build a database that could provide a benchmark for farm performance, help future researchers, and offer a reference for future policies [138]. The field of application for smartphones is vast, and this technology may have the most potential and benefits compared to other PLF tools, as mentioned earlier. It can be applied as a sensor to classify cattle’s rumination behaviors and grass intake, with accuracy over 90% [28]. Xu et al. [139] evaluated a method for cattle face recognition that had 91.3% accuracy. Farm management has been the focus of a number of studies, such as Vittis and Kaler [140], observed its relationship with lameness and found that it can be a contributor if the ongoing management of many factors is not carried out. Meanwhile, Belanche et al. [141] evaluated a smartphone-based technology approach to improving farm productivity (Table 3). Some scholars predict that smartphone-based technology will allow farmers to perform tests and obtain quick results on-site [114].

Smartphones might be the most versatile and accessible devices. By leveraging their integrated properties, diverse applications, and easy access to information, even small farmers can embrace PLF.

**Table 3:** Studies on behavior and activity detection for different livestock species using smartphones.

Species	Aim	Technology	Livestock System	Country	Main Result	Reference
Cattle	Evaluation of CattleFaceNet in cattle’s face recognition	RetinaFace–MobileNet for face detection and location, and ArcFace	Intensive	China	Accuracy of 91.3% in face identification	[139]
	Classifying cattle’s rumination behaviors and grass intake, based on data collected from a smartphone	Smartphone (iPhone 4S), fitted to cows in a halter	Semi-extensive	Belgium	Accuracy of 92% in detecting grass intake and ruminating behaviors	[28]
Goat	Evaluation of the efficiency of the Eskardillo tool, in managing farm production	Eskardillo (an Android smartphone-based terminal)	Intensive	Spain	The farms in question reduced their unproductive and dry period lengths	[141]
Sheep	Study the effects of farm management and conditions on sheep’s lameness	Lameness smartphone application	Semi-extensive	United Kingdom	Lameness can be caused by many factors	[140]

The number of animals in the research varied between 12 and 19 and the farm between 2 and 18.

## 6. Virtual Fencing

Virtual fencing for animals is an innovative technology concerning methods of animal containment and control. Virtual boundaries for animals are created using GPS technology and complex algorithms instead of physical ones [142]. Animals wear GPS-enabled collars or tags that communicate with a centralized system [143]. To force animals to stay in designated areas or prevent them from crossing boundaries, virtual fence systems can use stimuli such as noise, vibration, or mild electrical pulses [144]. These act as humane deterrents and ensure that the animal learns to associate certain cues with boundaries [142]. In PLF, virtual fencing is a pivotal component in optimally managing animals by enhancing the efficiency, welfare, and resource utilization [142]. As virtual fencing continues to be developed, it could lead to the reshaping of how we manage and interact with animals in various contexts.

## 7. Advantages and Limitations of Monitoring Devices

PLF has witnessed significant advancements with the integration of wearable devices, cameras, drones, and smartphones, each offering distinct advantages and facing certain limitations, as illustrated in Table 4. Balancing the advantages and overcoming the limitations is essential in maximizing the potential of these technologies in the dynamic landscape of PLF. Certain limitations can be overcome by combining different devices; this would enhance its capabilities, allowing innovative solutions for various problems, which some of the studies listed in Table 1 have considered in order to increase the accuracy of addressing a precise aspect.

Another example of how different sensors can assist each other is found in the experiment of Li et al. [145], who monitored ruminants in vast pastures using drones and GPS collars. As GPS data helped to detect the targeted animals’ locations, drones were deployed within the centroids of animal clusters, and whenever the GPS data were updated, the locations of deployments followed suit. This combination helped to cover most of the animals, although the distance between the drones and animals was lower than average.

Analyzing PLF data requires an organized approach to derive valuable information for decision making. It includes defining objectives and key performance indicators, understanding data sources, assessing data quality (ensuring that sensors and monitoring devices are calibrated correctly), aggregating and integrating data from various sources, normalizing and visualizing data (such as charts, graphs, and dashboards to represent the data in a clear and easily understandable manner), conducting time-series and correlation analysis (exploring correlations between different variables), utilizing statistical analysis techniques to identify significant relationships in the data, implementing alerts and anomaly detection, using the analyzed data as a foundation for decision support, establishing a feedback loop for continuous improvement, and prioritizing training and capacity building. This process can ensure that PLF data are leveraged strategically to help to make decisions and enhance farm management.

**Table 4:** Advantages and limitations of precision livestock technologies.

	Advantages	Limitations
**Wearable devices**	Real-time monitoring [63] Non-invasive [146] Reduces labor [147] Remote monitoring [147] Data-driven decision making [88]	Battery life [21] Cost [148] Device attachment problems [35] Ethical considerations [147]
**Cameras**	Real-time monitoring [69] Non-invasive [146] Capture detailed data [62]	Environmental conditions [146] Limited field of view [62] Cost [146]
**Drones**	Reduced labor [149] Aerial perspective [121] Efficient data collection [149] Flexibility in terrain coverage [149]	Limited flight time [150] Weather dependence [151] Restricted payload capacity [150] Risk of disturbance [62] Cost [149]
**Smartphone**	Portable [152] Cost-effective [152] Integrated with sensors [152] Data storage and visualization [152]	Battery life [28] Data security [134] Limited processing power [153]

## 8. Conclusions

In this review, we aimed to discuss the status of a set of precision technologies that have an important role in increasing production and assess their continuous growth and evolution. Several studies discussed above achieved huge success in their tasks, while others showed great potential. It seems obvious from this review that the understanding of technological development by farmers is crucial for the better management of livestock and natural resources.

Some other aspects that need to be addressed and are of extreme importance are temperature analysis using thermal sensors; metabolic data collection using different methods, such as heart rate monitoring, respiration sensors, or ingestible sensors to track digestion from within the animal; and hormone evaluation using specialized sensors or conducting blood and saliva tests regularly. The future of PLF needs to be shaped to be more inclusive, sustainable, and capable of meeting the evolving needs of the farming industry. Some of the key considerations include affordability and accessibility, user-friendly technology, and ethical concerns. Integrating artificial intelligence and machine learning could also lead to further development. One obvious observation that can explain the reluctance of an important number of farmers to invest in these smart tools is the high price and the lifetime of some devices, whose batteries are not replaceable.

## Data Availability

Not applicable.
